# Rapid *in vitro* production of single-stranded DNA

**DOI:** 10.1093/nar/gkz998

**Published:** 2019-11-12

**Authors:** Dionis Minev, Richard Guerra, Jocelyn Y Kishi, Cory Smith, Elisha Krieg, Khaled Said, Amanda Hornick, Hiroshi M Sasaki, Gabriel Filsinger, Brian J Beliveau, Peng Yin, George M Church, William M Shih

**Affiliations:** 1 John A. Paulson School of Engineering and Applied Sciences, Harvard University, Cambridge, MA 02138, USA; 2 Wyss Institute for Biologically Inspired Engineering at Harvard University, Boston, MA 02115, USA; 3 Department of Cancer Biology, Dana-Farber Cancer Institute, Boston, MA 02215, USA; 4 Department of Biological Chemistry and Molecular Pharmacology, Harvard Medical School, Boston, MA 02115, USA; 5 Department of Systems Biology, Harvard Medical School, Boston, MA 02115, USA; 6 Department of Genetics, Harvard Medical School, Boston, MA 02115, USA

## Abstract

There is increasing demand for single-stranded DNA (ssDNA) of lengths >200 nucleotides (nt) in synthetic biology, biological imaging and bionanotechnology. Existing methods to produce high-purity long ssDNA face limitations in scalability, complexity of protocol steps and/or yield. We present a rapid, high-yielding and user-friendly method for *in vitro* production of high-purity ssDNA with lengths up to at least seven kilobases. Polymerase chain reaction (PCR) with a forward primer bearing a methanol-responsive polymer generates a tagged amplicon that enables selective precipitation of the modified strand under denaturing conditions. We demonstrate that ssDNA is recoverable in ∼40–50 min (time after PCR) with >70% yield with respect to the input PCR amplicon, or up to 70 pmol per 100 μl PCR reaction. We demonstrate that the recovered ssDNA can be used for CRISPR/Cas9 homology directed repair in human cells, DNA-origami folding and fluorescent *in*-*situ* hybridization.

## INTRODUCTION

DNA is instrumental to myriad applications in biological imaging, bionanotechnology and synthetic biology. Many of these applications rely on the availability of ssDNA. Depending on the required size, scale and purity, the production of ssDNA can become prohibitively expensive or onerous. Although chemically synthesized ssDNA is commercially available and declining in cost, such ssDNA is limited beyond lengths of ∼200 nt (1) and requires additional processing to remove impurities. Alternatively, production of single strands from a double-stranded DNA (dsDNA) template via enzymatic processing (2), micro-bead sequestration (3), rolling circle amplification (4,5), asymmetric polymerase chain reaction (PCR) (6) and co-polymerization and electrophoresis methods (7,8) may be used, but is frequently limited by complexity of the protocols, scalability and/or purity of the recovered strands. Recently, Palluk *et al.* demonstrated a novel enzymatic approach for the *denovo* synthesis of ssDNA (9). However, this method has not yet been used to synthesize strands longer than 10 nt at high yield. Similarly, autonomous ssDNA synthesis via primer exchange reaction (PER) is currently limited to lengths of 60 nt (10). Phagemid *in vivo* production of ssDNA can generate biotech-scale quantities of arbitrary sequences, but the method is less amenable to rapid prototyping due to increased lag time between sequence design and strand production (11). Our lab previously developed Selective Nascent Polymer Catch-and-Release (SNAPCAR) allowing production of long ssDNA up to 7kb (12); however, the protocol to recover strands was lengthy and cumbersome. Each time PCR is performed for a desired strand, the polymer must be grown under low-oxygen conditions, posing a nuisance for standard molecular-biology laboratories to adopt the methodology (12,13). Thus, a need persists for methods allowing fast (ideally within 2–3 h), user-friendly, high-yielding and low-cost *in vitro* production of ssDNA longer than 200 nt.

## MATERIALS AND METHODS

### Solvents and reagents for MeRPy-PCR

All solvents and reagents were purchased from commercial vendors. Methanol (Sigma-Aldrich, 322415), Isopropanol (Fisher Scientific, A426P-4), 1,2-Dimethylethylenediamine (Sigma-Aldrich, D157805-5G), Molecular biology grade glycogen (Thermo Fisher, R0561), Molecular biology grade acrylamide 40wt% (Sigma-Aldrich, 1697–500ML), Sodium acrylate (Sigma-Aldrich, 408220-35G), Molecular biology grade tetramethylethylenediamine (Life Technologies Corp, 15524010), Ammonium persulfate (Sigma, A3678-25G). All acrylamide-labeled oligonucleotides and ultramer/megamer ssDNA was purchased from Integrated DNA Technologies. All double-stranded DNA templates used were purchased from Twist Bioscience. Taq DNA polymerase (NEB, M0273L), Phusion (NEB, M0531S).

### UV/Vis absorbance

Nanodrop 2000c Spectrophotometer (Thermo Scientific) was used to record the data.

### Agarose gel electrophoresis (AGE)

UltraPure agarose (Life technologies, 16500500) was used to prepare agarose gels of various percentages. DNA Origami structures were eluted for 3 h at 60 V in pre-stained Ethidium bromide (Bio Rad, 1610433) gels in 0.5 × Tris-borate-EDTA (TBE) buffer containing 11 mM MgCl_2_. Double-stranded and single-stranded DNA was eluted in 0.5 × TBE buffer at 150 V for 1–1.5 h (depending on the size of the oligo and percentage of gel). Gels were either pre-stained with Ethidium bromide (Bio Rad, 1610433) or post-stained with SYBR Gold (Thermo Fisher, S-11494).

### Denaturing and native polyacrylamide gel electrophoresis (dPAGE and nPAGE)

UreaGel System (National Diagnostics, EC-833-2.2LTR) was used to prepare denaturing polyacrylamide gels of various percentages. Forty wt% acrylamide (Fisher Scientific, BP1406-1) was used to prepare native polyacrylamide gels of 20%. Gels were eluted at 200–300 V for 20–40 min (depending on the size of the oligo and percentage of gel) and post-stained with SYBR Gold (Thermo Fisher, S-11494) for 20 min before imaging. Densitometry analysis of DNA bands was performed with ImageJ (v2.0.0-rc-69/1.52i) (14).

### Transmission electron microscopy (TEM)

A total of 3 μl of the crude DNA origami folding reaction diluted 1:10 in identical buffer conditions was applied to a FCF400-CU-50 transmission electron microscopy (TEM) grid (Fisher Scientific, 5026034) and incubated for 2 min, followed by 3 μl of 2% uranyl formate solution containing 25 mM NaOH, incubation of 1–2 s and immediate removal with filter paper (Fisher Scientific, 09-874-16B) and air drying. Imaging was performed at 80 kV on a JEOL JEM 1400 plus.

### Polymerase chain reaction (PCR) and DNA origami folding

A PTC-225 Peltier Thermal Cycler (MJ Research) in conjunction with various thermocycler protocols.

### Metaphase DNA FISH

The metaphase DNA FISH protocol was developed from refs. (15–17). Human metaphase chromosome spreads (XX 46N or XY 46N, Applied Genetics Laboratories) were denatured in 2 × SSC + 0.1% (vol/vol) Tween-20 (SSCT) + 70% (vol/vol) formamide at 70°C for 90 s before being immediately transferred to ice-cold 70% (vol/vol) ethanol for 5 min. Samples were then immersed in ice-cold 90% (vol/vol) ethanol for 5 min and then transferred to ice-cold 100% ethanol for a further 5 min. Slides were then air-dried before 25 μl of ISH solution comprising 2 × SSCT, 50% (vol/vol) formamide, 10% (wt/vol) dextran sulfate, 40 ng/μl RNase A (EN0531, Thermo Fisher) and MeRPy-PCR generated probe pool at 1.5 μM final concentration was added. Rubber cement was used to seal the hybridization solution underneath a coverslip, and the sample was placed into a humidified chamber inside an air incubator at 45°C overnight. After hybridization, samples were washed in 2 × SSCT at 60°C for 15 min and then in 2 × SSCT at room temperature (2 × 5 min). Samples were then mounted with 12 μl of SlowFade Gold + DAPI (Thermo Fisher S36939) and sealed underneath a coverslip with nail polish before imaging.

### Microscopy

Imaging of iterative branching samples was conducted on an inverted Zeiss Axio Observer Z1 using a 100× Plan-Apochromat Oil N.A. 1.40 objective. Samples were illuminated by using Colibri light source using a 365 nm or 555 LED. A filter set composed of a 365 nm clean-up filter (Zeiss G 365), a 395-nm long-pass dichroic mirror (Zeiss FT 395) and a 445/50 nm band-pass emission filter (Zeiss BP 445/50) was used to visualize DAPI staining. A filter set composed of a 545/25-nm excitation filter (Zeiss BP 545/25), a 570 nm long-pass dichroic mirror (Zeiss FT 570) and a 605/70 nm band-pass emission filter (Zeiss BP 605/70) was used to visualize Cy3 signal. Images were acquired by a Hamamatsu Orca-Flash 4.0 v3 sCMOS camera with 6.5 μm pixels, resulting in an effective magnified pixel size of 65 nm.

### DNA synthesis and purification

The 42k library targeting Human Chromosome 8 was ordered from Twist Bioscience and emulsion PCR was performed as previously in ref.’s (15–17). This emulsion PCR product was diluted to a final concentration of 1.25 pg/μl for subsequent amplification and ssDNA recovery with the MeRPy-PCR protocol containing a Cy3-labeled reverse primer ordered from Integrated DNA Technologies.

### Cas9 directed HDR in human cells with ssDNA

Human Embryonic Kidney (HEK) 293Ts with a broken GFP expression vector with AAVS1 gRNA targets were obtained from the Church lab that were negative for mycoplasma infection. They were expanded using 10% fetal bovine serum in high-glucose DMEM with glutamax passaging at a typical rate of 1:100 and maintained at 37°C with 5% CO_2_. Transfection was conducted using Lipofectamine 2000 (Thermofisher Catalogue # 11668019) using the protocol recommended by the manufacturer with slight modifications outlined below. Twenty-four hours before transfection ∼5.0 × 10^4^ cells were seeded per well in a 24-well plate along with 0.5 ml of media. A total of 1 μg of plasmid DNA was transfected using 2 μl of Lipofectamine 2000 per well. The DNA content per well contained 700 ng of hCas9 mixed with 200 ng of gRNA expressing plasmid and 100 ng of ssDNA donor (0.76 pmol for 200 bp donor). Homology directed repair (HDR) was measured by percentage of GFP+ through FACS as follows. Three days post-transfection, the cells were harvested using TrypLE and strained before analysis on the BD LSR. Live cell population was gated using SSC and FSC to separate debris and singlets. GFP+ gates were set using a transfected control cell population that did not receive the HDR donor and controls were performed with ssDNA oligo donor transfection alone.

## RESULTS AND DISCUSSION

**Figure 1.**
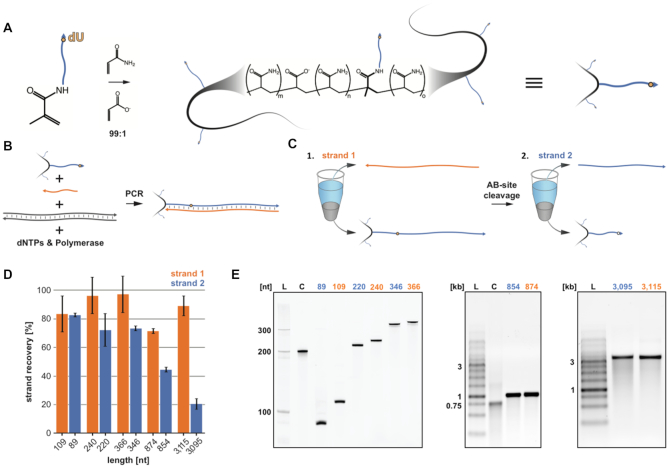
MeRPy-PCR overview and recovery yields for strands 1 (untagged) and 2 (initially tagged) of different amplicon lengths. (**A**) Production of the polymer-tagged primer. A 5′ acrydite modified primer is polymerized with acrylamide and sodium acrylate (ratio 99:1) to form a long linear DNA-tagged polymer. (**B**) MeRPy-PCR procedure following standard PCR guidelines. (**C**) (1.) Recovery of strand 1 under alkaline denaturing conditions and methanol precipitation. (2.) Recovery of strand 2, after treatment with UDG and DMEDA followed by a methanol precipitation. (**D**) Recovery yield for strand 1 and 2 of various lengths. Bar graphs denoting the recovery yield (%). Strand recovery yield was determined by the absolute recovered strand yield (pmol) relative to MeRPy-PCR input (pmol). Data are shown as mean ± STD (*N* = 3). (**E**) Gel electrophoresis of MeRPy-PCR derived ssDNA. Left, denaturing polyacrylamide gel with L – 20 bp Ladder, C – 200mer control from Integrated DNA Technologies (IDT). Middle and right, native agarose gels with L – 1 kb Ladder, C–750mer control from IDT. MeRPy-PCR derived and commercial ssDNAs were loaded with normalized mass amounts for each gel lane in (E).

We present an updated method that we call Methanol-Responsive Polymer PCR (MeRPy-PCR). We create a set of primers bearing a linear polyacrylamide-co-acrylate tag by co-polymerizing a 5′ acrydite-modified primer with acrylamide and sodium acrylate (Figure 1A; Supplementary Figure S1 and Tables S1–2; and Protocol 1). The modified primer can include a deoxyuridine (dU), which can be placed anywhere along the sequence and allows the site-specific creation and subsequent cleavage of an abasic site (AB-site). We use the polymer-tagged primer in an otherwise standard PCR reaction, resulting in a polymer-tagged amplicon (Figure 1B and Supplementary Protocol S2) that subsequently allows the selective precipitation and recovery of both forward and reverse strands away from each other (Figure 1C and Supplementary Figure S2). Substitution of a polymer-tagged primer had no noticeably adverse effects on PCR in terms of strand yield and purity (Supplementary Figure S3). Furthermore, the MeRPy primer can easily be grown in bulk (standard preparation scale is 5 ml at 5–10 μM) eliminating the need for frequent polymerization.

After PCR, we first recover untagged strand 1 in a supernatant by performing a denaturing precipitation under alkaline conditions by addition of NaOH to 44 mM final concentration, followed by mixing with one equivalent of methanol and then centrifugation at 350–2000 RCF (Figure 1C1). We next recover complementary strand 2 by resuspending the precipitated polymer-DNA pellet and incubating it with uracil-DNA glycosylase (UDG) for 15 min to excise the dU nucleobase and create an AB-site. The AB-site is then cleaved by incubating the polymer-DNA solution with 100 mM dimethylethylenediamine (DMEDA) (18) for 15 min, followed by precipitation in 50% methanol to remove the waste polymer-tagged DNA (Figure 1C2). The entirety of this procedure takes ∼80–90 min (depending on strand amplicon length and not including the time it takes to run the PCR), with strand 1 recovery accounting for the first ∼40–50 min (Supplementary Figure S2).

We used this method to generate ssDNA ranging from 89–3115 nt in length by amplifying an array of target sequences with MeRPy-PCR and recovering both strands 1 and 2 of each amplicon (Figure 1D; Supplementary Figure S4 and Note S1). The strand-recovery protocol was nearly identical for all lengths and templates, apart from slight differences in the alkaline denaturation step for the longest amplicons (see Supplementary Protocol S3). Strand 1 was routinely recovered with a yield of 70% to >90% with respect to the initial MeRPy-PCR amplicon. By contrast, recovery yield of strand 2 was lower as the length of the amplicons increased (see Supplementary Note S2). We recorded absolute yields of ∼2.2–12 pmol/100 μl (0.31–2.49 μg/100 μl) PCR for strand 1 and ∼0.5–12 pmol/100 μl (0.29–0.97 μg/100 μl) PCR for strand 2 (Supplementary Figure S5 and Yield Data). It should be noted that the final amount and purity of recovered ssDNA depends on the efficiency and cleanliness of the PCR, therefore PCR optimization may be desirable. Furthermore, we observed that ssDNAs recovered from MeRPy-PCR were of high purity, comparable to SNAPCAR produced ssDNA and on par or better than a chemically-synthesized 200mer oligonucleotide after PAGE purification and an enzymatically produced 754mer oligonucleotide purchased from the commercial vendor Integrated DNA Technologies (Figure 1E). To demonstrate the utility of MeRPy-PCR generated ssDNA for demand-meeting applications, we show CRISPR/Cas9-mediated HDR in human cells, fluorescent *in*-*situ* hybridization (FISH) imaging and DNA-origami folding. We picked the untagged strand 1 for each application, based on the higher overall recovery yield and briefer protocol. Each of the three tested applications utilizes ssDNA in varying capacities; DNA origami requires long ssDNA scaffolds (>1 kb, 0.5 pmol for a 50 μl folding reaction) (19–21), FISH requires a library of >100 nt Cy3-labeled strands at ∼100–200 pmol per experiment to improve sensitivity when tiling specific regions of the genome (15), and CRISPR/Cas9 directed HDR has seen growing interest in the field to use long ssDNA over dsDNA donors (22–24), which can be difficult to produce or else prohibitively expensive to purchase at sufficient scale (10–12 pmol for one triplicate experiment) for cell-culture experiments.

**Figure 2.**
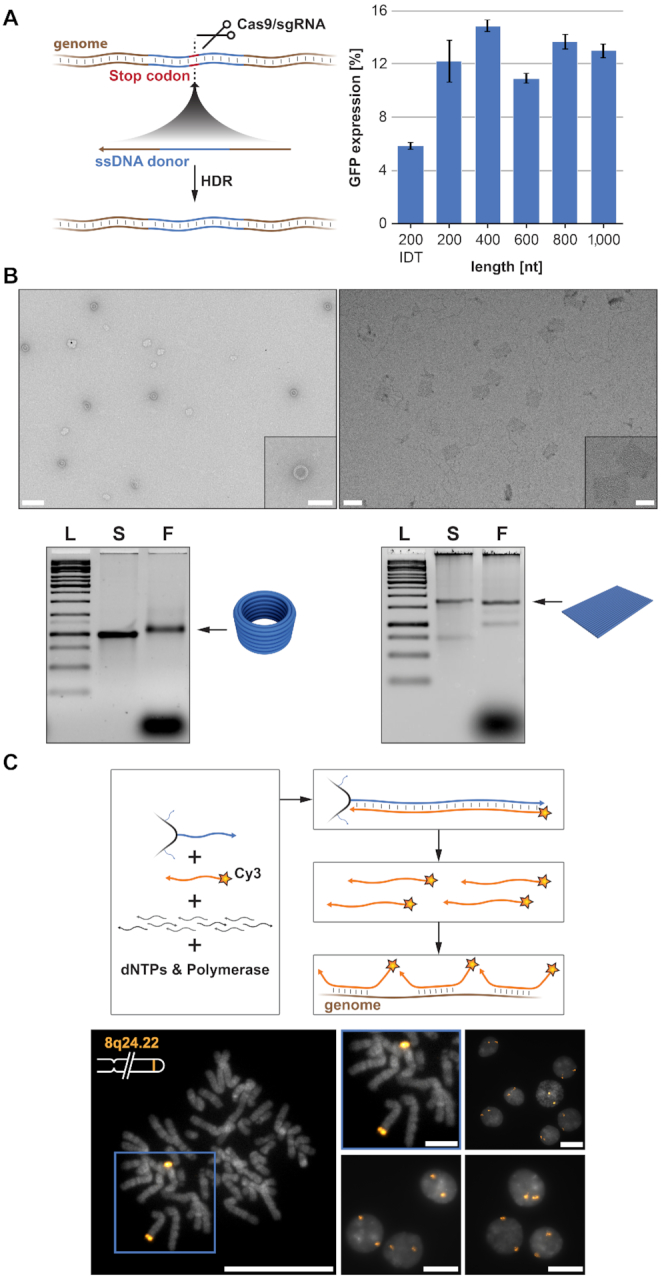
Applications using ssDNA of various lengths. (**A**) Genome editing in human cells using CRISPR/Cas9. (left) A genomically integrated GFP coding sequence is disrupted by the insertion of a stop codon and a 68-bp genomic fragment from the AAVS1 locus. Restoration of the GFP sequence by HDR with a ssDNA donor sequence results in GFP+ cells that can be quantified by FACS. (right) Bar graph depicting HDR efficiencies induced by MeRPy-PCR derived ssDNAs of different lengths versus a 200mer chemically synthesized strand from IDT. Data are shown as mean ± STD (*N* = 3). (**B**) ssDNA scaffold was generated via MeRPy-PCR from the phage genome, p7308 and used in the folding of a 30 nm DNA origami barrel and a 20 nm rectangle. Agarose gel electrophoresis shows the 1 kb Ladder (L), purified scaffold strand (S) alongside the folded barrel and 20 nm rectangle structures (F). TEM depicts the folded DNA origami barrel (left) and a 20 nm rectangle (right). Scale bars denote 100 and 50 nm for small insert. (**C**) A library comprising 42 000 probe sequences designed to tile along an 8.4 Mbp region of Human Chromosome 8 was amplified from a small amount of template using MeRPy-PCR with a Cy3-labeled reverse primer and subsequent recovery of fluor-tagged strand 1 library. The generated fluor-labeled ssDNA library was validated *insitu* on fixed human metaphase spreads and interphase cells. Scale bars denote 20 μm (zoom of metaphase spread scale bar denotes 5 μm).

For HDR, we assessed the performance of MeRPy-PCR generated ssDNA donors (Supplementary Figure S6) of varying size, relative to a purchased chemically synthesized 200 nt donor from IDT. The ssDNA donor-mediated HDR removed a stop codon from a broken GFP expression vector, restoring the GFP sequence and expression (Figure 2A; Supplementary Figure S6 and Note S3). We generated five different ssDNA donors from 200 to 1000 nt, only varying the homology arm length. We produced the ssDNA donors at yields of ∼13–34 pmol/100 μl (1.77–4.15 μg/100 μl) PCR (Supplementary Table S3). The efficiency of HDR was comparable for the different MeRPy-PCR generated ssDNA and was on par with the 200 nt chemically synthesized donor.

Next, we tested the ability to produce custom scaffolds for DNA-origami folding. DNA origami is often limited to a defined number of ssDNA scaffolds based on the availability of different M13 phage genomes. There is growing interest in the field for the design and production of new scaffolds that offer a larger range of sequence space (25). We tested MeRPy-PCR derived ssDNA by folding a 30 nm DNA-origami barrel (26) with a 3315 nt scaffold and a 20 nm rectangle with a 7308 nt scaffold, both generated from a p7308 M13 genome (Figure 2B, Supplementary Figure S7 and Protocol S4). We successfully produced the 3315 nt scaffold with 1.35 pmol/100 μl (1.37 μg/100 μl) and the 7308 nt scaffold with 1 pmol/100 μl (2.23 μg/100 μl) PCR (Supplementary Table S3).

At last, we demonstrated the ability to use MeRPy-PCR to generate a large library of FISH probes with a Cy3-modified primer (Figure 2C). We generated ∼70 pmol/100 μl (2.81 μg/100 μl) PCR of Cy3-modified ∼130 nt FISH probes (Supplementary Table S3), that can successfully be used in imaging a distinct locus of the genome (Chromosome 8q24.22) (17). As expected by the FISH probe design, we observed two puncta per cell, with both puncta located toward the end of two similarly sized, medium-length chromosomes (Figure 2C). Use of MeRPy-PCR here highlights the ease with which FISH probes with Cy3 modifications can be generated in sufficient quantities for imaging, obviating the need for expensive and time-consuming purifications or conjugation chemistries.

In summary, we have demonstrated that MeRPy-PCR can be performed without the need for additional optimization beyond that needed for PCR in general, and can be used to recover high yields of both forward and reverse strands, the latter with a briefer protocol and higher yields. We further demonstrated that the generated ssDNA can be used in a variety of demand-meeting applications in synthetic biology, bionanotechnology and biological imaging. The short time frame to recover the strands is user-friendly and lowers the bar to rapid in-house production of large quantities of ssDNA. Importantly, the low-cost production (further cost reduction could potentially be achieved via in-house production of *Taq* polymerase) of strands via MeRPy-PCR may enable the accelerated exploration of scaffold design space in DNA origami, of genome visualization with FISH, and of the efficiency and off-target effects of single stranded donor DNA in CRISPR/Cas9-mediated HDR.

## Supplementary Material

gkz998_Supplemental_Files

## Notes

Present address: Elisha Krieg, Leibniz-Institut für Polymerforschung Dresden e.V., Germany; Faculty of Chemistry and Food Chemistry, Technische Universität Dresden, Germany.

Present address: Brian J. Beliveau, Department of Genome Sciences, University of Washington, Seattle, WA, USA.
