# Supplementary material for: Rapid in vitro production of single-stranded DNA
Source: Nucleic Acids Res. 2019 Nov 12;47(22):11956–62. doi: 10.1093/nar/gkz998 (PMC7145709; doi:10.1093/nar/gkz998)
Supplement: gkz998_Supplemental_Files [file gkz998_supplemental_files.zip › 191028_MeRPy_Supporting_Information.pdf]

Supporting Information

Title

Rapid *in vitro* production of single-stranded DNA

Authors

D. Minev[1,2,3,4,†], R. Guerra[2,3,4,†], J. Y. Kishi[2,6,‡], C. Smith[2,5,‡], E. Krieg[2,3,4,||], K. Said[2,5], A. Hornick[2,5], H. M. Sasaki[2,6], G. Filsinger[2,5], B. J. Beliveau[2,6,§], P. Yin[2,6], G. M. Church[2,5], and W. M. Shih[2,3,4,*]

Affiliations

[1]John A. Paulson School of Engineering and Applied Sciences, Harvard University, Cambridge, Massachusetts, USA;

[2]Wyss Institute for Biologically Inspired Engineering at Harvard University, Boston, Massachusetts, USA;

[3]Department of Cancer Biology, Dana-Farber Cancer Institute, Boston, Massachusetts, USA;

[4]Department of Biological Chemistry and Molecular Pharmacology, Harvard Medical School, Boston, Massachusetts, USA;

[5]Department of Genetics, Harvard Medical School, Boston, Massachusetts, USA;

[6]Department of Systems Biology, Harvard Medical School, Boston, Massachusetts, USA;

[†]denotes equal contribution

[‡]denotes equal contribution

[||]Present Address: Leibniz-Institut für Polymerforschung Dresden e.V., Germany; Faculty of Chemistry and Food Chemistry, Technische Universität Dresden, Germany;

[§]Present Address: Department of Genome Sciences, University of Washington, Seattle, Washington, USA;

*Correspondence: William_Shih@dfci.harvard.edu

Table of Contents

Protocols **3**

Protocol S1: MeRPy-primer polymerization 3

Part I: Polymerization procedure 3

Part IIa: Purification procedure (for tagged primer - TP) 5

Part IIb: Purification procedure (for polymer without primer - P) 6

Protocol S2: General PCR setup 7

Protocol S3: Strand recovery 10

Part I: Strand 1 recovery (native and denaturing precipitations) 10

Part II: Strand 2 recovery (UDG/DMEDA strand cleavage) 11

Part III: Isopropanol precipitation 12

Protocol S4: DNA origami folding 13

Supplemental Figures **15**

Supplemental Tables **20**

Supplemental Notes **22**

References **31**

Protocols

Protocol S1: MeRPy-primer polymerization

Materials

- Single-stranded DNA primer with 5´ acrydite modification (Avoid excessive light exposure)
- Acrylamide (AA) (40 wt% stock solution, stored light protected at 4°C)
- Sodium acrylate (SA) (20 wt% stock solution, stored light protected at 4°C)
- 5x TBE buffer; 500 mM Tris, 500 mM boric acid,10 mM EDTA, pH 8.2
- 10x TE buffer, 50 mM Tris, 1 mM EDTA, pH 8.0
- 5 M NaCl
- Tetramethylethylenediamine (TEMED, stored light protected at 4°C)
- Ammonium persulfate (APS, protected from moisture)
- Nitrogen gas (N_2)
- Hypodermic needle
- 2 mL glass vial with septum cap
- Methanol (MeOH), stored at -20°C
- Filtered ddH_2O
- 50 mL centrifuge tubes
- 20 mL disposable syringe

Part I: Polymerization procedure

Please see the supplementary video to better follow the written protocol of the polymerization procedure (https://www.youtube.com/watch?v=CxIgF7SMi3I).

Note: If changing the production scale, ensure that the reaction container (glass vial) has the appropriate size: to achieve efficient N_2 purging, 1/4 to 2/3 of the container volume should be filled with the reaction solution. Prepare the following reactions according to the steps listed below:

Note: Polymerization of the polymer-tagged primer with acrylamide and sodium acrylate (ratio 99:1) is necessary to reduce co-precipitation of free single-stranded DNA (primer) after PCR (see ref (1) for more information).

Sample:	H2O [μL]:	TBE, 5x [μL]:	Acrydite tagged primer [μL]:	Acrylamide (AA), 40% [μL]:	Sodium acrylate (SA), 20% [μL]:	TEMED, 5% [μL]:	APS, 5wt% [μL]:	Total volume [μL]:
TP	285.25	100	50	62.5	1.25	0.5	0.5	500
P	335.25	100	-	62.5	1.25	0.5	0.5	500

Final concentrations in the reaction:

Sample:	Final AA concentration [%]:	Final SA concentration [%]:	Final APS/TEMED concentration [wt%]:	Initial concentration of acrydite tagged primer [μM]:	Final concentration of acrydite tagged primer [μM]:
TP	5	0.05	0.005	1,000	100
P	5	0.05	0.005	-	-

TP – Polymer tagged with primer ssDNA (MeRPy-primer)

P – Polymer only

Steps

1. Add to glass vial: H_2O, TBE, acrydite tagged primer, AA and SA, then purge solution 20 minutes with N_2. **Note:** Pierce the septum cap with the hypodermic needle and make sure the cap is slightly open for slow N_2 bubbling.
    I. In the meanwhile, prepare fresh 5 wt% stock solutions of TEMED and APS:
        A. 5% TEMED: 6.5 μL TEMED + 93.5 μL H_2O
        B. 5 wt% APS: 25 mg APS + 0.5 mL with H_2O

2. Add TEMED to reaction, swirl vial, then add APS while continuing N_2 purging for 15 minutes.
3. Close septum cap tightly (stops the bubbling) and leave sample attached under nitrogen pressure overnight (keep nitrogen on, while cap is tightly closed).
    I. Sample should be highly viscous the next day.

- **Pause point:** Samples can be stored at 4°C and purification can be continued at a later date.

Part IIa: Purification procedure (for tagged primer - TP)

1. Add 4.5 mL 1X TE to viscous polymer solution:

    I. Add 350 µL 1X TE to the vial. Vortex for 1–2 minutes.
    II. Fill a 20 mL disposable syringe with air, attach a needle to the syringe and bend the needle to a ~90° angle, using the needle cover.
    III. Turn vial above 50 mL centrifuge tube upside down. Use the syringe to push in air into the bottom of the vial, so that the viscous solution is pushed out and drips into the centrifuge tube.
    IV. Add 0.5 mL 1X TE buffer to the emptied vial, vortex for 30 seconds, then repeat step III.
    V. Repeat step IV. 3 times. Make sure all of the polymer is washed out of the vial and into the 50 mL centrifuge tube.
    VI. Add another 2.15 mL 1X TE buffer to the centrifuge tube to get to a total volume of 4.5 mL added 1X TE buffer.

2. Vortex or shake at high speed for 10 minutes.
3. Add 25 µL 5 M NaCl and vortex for several seconds.
4. Take out 50 µL aliquot for analysis later (**UP**).
5. Use a syringe: Fill up with 5 mL cold MeOH. **Note:** remove the cap from the 50mL centrifuge tube and vortex on low speed with no cap, in order to avoid spillage.

    I. Slowly drip 5 mL MeOH, while vortexing on low speed. The last 1–2 mL of MeOH should turn the clear liquid turbid, forming a precipitate.

6. Let the sample incubate for 2 minutes on ice.
7. Centrifuge at 150 g for 5 minutes at 4°C and decant supernatant. There should be a visible pellet.

    I. Keep 100 µL of supernatant for later analysis (**SN1**).

8. Re-precipitate:

    I. Re-suspend pellet by adding 4.5 mL with H_2O, vortex for 10 minutes (make sure the pellet is perfectly dispersed), then add 0.5 mL 10X TE buffer and 30 µL 5 M NaCl.

II. Use a syringe: Fill up with 5 mL cold MeOH.
   a. Slowly add 5 mL, while vortexing. The last 1–2 mL of MeOH should turn the clear liquid turbid, forming a precipitate.

III. Centrifuge again at 150 g for 5 minutes at 4°C and decant supernatant.

IV. Keep 100 µL of supernatant for later analysis (**SN2**).

9. Add 4.5 mL H_2O to the pellet and vortex for 10 minutes. Make sure the pellet is perfectly dispersed.
10. Add 0.5 mL 10X TE buffer (final concentration of 0.5 wt% polymer tagged with primer ssDNA) and vortex shortly.
11. Divide into aliquots, and store at -20°C (**TP**).

Part IIb: Purification procedure (for polymer without primer - P)

Steps 1. to 8. are identical. **Note:** the unpurified polymer reaction (UP) and the supernatants 1 (SN1) and 2 (SN2) are not needed for analysis, since there is no DNA present in this sample.

9. Add 2.5 mL H_2O (final concentration of 1 wt% polymer without DNA) to the pellet and vortex for 10 minutes. Make sure the pellet is perfectly dispersed.
10. Divide into aliquots, and store at -20°C.

Protocol S2: General PCR setup

Here we report the generalized PCR mixes and thermocycler protocols for standard Taq polymerase as well as Phusion high-fidelity PCR master mix with HF buffer. These two polymerases generated all the ssDNA needed to carry out the experiments in this report. We also successfully performed the strand purification protocol from PCRs of different polymerases (Hot Start Taq DNA Polymerase and Kapa Taq), highlighting the generalizability of the recovery method. The results for Hot Start Taq DNA Polymerase and Kapa Taq are not reported here as the ssDNA generated was not a part of any downstream application or figure reported.

Standard Taq polymerase PCR setup

All PCRs using standard Taq polymerase were prepared using this general protocol:

**Component**	**100 μL reaction**	**Final concentration**
Nuclease-free H_2O	Bring volume up to 100 μL	
10X Standard Taq buffer	10 μL	1X
10 mM (each) dNTPs	2 μL	200 μM
10 μM untagged primer	2 μL	0.2 μM
X μM MeRPy-primer	Variable*	0.2 μM
10 nM DNA template	1 μL	0.1 nM
Standard Taq DNA polymerase	0.5 μL	2.5 units/100 μL PCR

*Amount of MeRPy-primer to use depends on stock concentration prepared after polymerization, but the final concentration should be equal to that of the untagged primer.

Standard Taq polymerase thermocycler protocol

Step	Temperature (°C)	Time (s)
Initial denaturing	95	30
Denaturing	95	15
Annealing	Variable*	15
Extension	68	Variable**
Cycle	X 29 additional cycles	
Final extension	68	300
Hold	4	forever

*The annealing temperature depends on the exact primer pair used. Annealing temperatures were calculated based on NEB's Tm calculator's recommended temperature (generally 5°C below the annealing temperature of the primer with the lower Tm).

**The extension temperature for all lower range amplicons (>500 nt) was 15 seconds, however higher range amplicons followed NEB's recommend extension time of 1 minute per kb.

Phusion PCR setup

Component	100 µL reaction	Final concentration
Nuclease-free H_2O	Bring volume up to 100 µL	
10 µM untagged primer	5 µL	0.5 µM
X µM MeRPy-primer	Variable*	0.5 µM
10 nM DNA template	4 µL	0.4 nM
2X Phusion master mix	50 µL	1 X

*Amount of MeRPy-primer to use depends on stock concentration prepared after polymerization, but the final concentration should be equal to that of the untagged primer.

Phusion thermocycler protocol

Step	Temperature (°C)	Time (s)
Initial denaturing	98	30
Denaturing	98	10
Annealing	Variable*	30
Extension	72	Variable**
Cycle	X 29–34 additional cycles	
Final extension	72	240
Hold	4	forever

*The annealing temperature depends on the exact primer pair used. Annealing temperatures were calculated based on NEB's Tm calculator's recommended temperature (generally at the annealing temperature of the primer with the lower Tm).
**The extension temperature followed NEB's recommend extension time of 15–30 seconds per kb.

KOD Hot Start PCR setup

Component	100 µL reaction	Final concentration
Nuclease-free H_2O	Bring volume up to 100 µL	
10X Standard Taq buffer	10 µL	1X
25 mM $MgSO_4$	7.2 µL	1.8 mM
2 mM dNTPs	10 µL	0.2 µM
10 µM untagged primer	5 µL	0.5 µM
X µM MeRPy-primer	Variable*	0.5 µM
10 ng/µL DNA template	1	0.1 ng/µL
KOD Hot Start DNA Polymerase	2	2 units/100 µL PCR

*Amount of MeRPy-primer to use depends on stock concentration prepared after polymerization, but the final concentration should be equal to that of the untagged primer.

KOD Hot Start thermocycler protocol

Step	Temperature (°C)	Time (s)
Initial denaturing	95	120
Denaturing	95	20
Annealing	Variable*	10
Extension	70	Variable**
Cycle	X 29 additional cycles	
Final extension	70	60
Hold	4	forever

*The annealing temperature depends on the exact primer pair used. Annealing temperatures were calculated based on NEB's Tm calculator's recommended temperature (generally at the annealing temperature of the primer with the lower Tm).
**The extension temperature followed Novagen's recommend extension time of 10-25 seconds per kb.

Protocol S3: Strand recovery

Materials

- 1 wt% linear polyacrylamide (without DNA primers) in H_2O
- Methanol (MeOH), stored at -20ºC
- Basic denaturing buffer (BDB; 0.2 M NaOH, 2 mM EDTA)
- Washing solution (1 part 1X Tris-EDTA, 30 mM NaCl and 1 part MeOH)
- Uracil DNA glycosylase (UDG) and accompanying 10X UDG buffer
- 10X 1,2-Dimethylethylenediamine (DMEDA; 1 M, adjusted to pH 8–9 with acetic acid)
- 1 M sodium chloride (NaCl)
- Isopropanol
- 3 M sodium acetate (NaOAc; pH 5.3)
- Molecular biology grade glycogen
- 70–75% ethanol (EtOH)
- 1X Tris-EDTA (TE)
- H_2O
- 1.5–2 mL centrifuge tubes

Note: Long vortexing is significantly easier with a Vertical 50 and 15 mL, Microtube Holder (Scientific Industries, Inc., SI-V525).

Part I: Strand 1 recovery (native and denaturing precipitations)

1. Add 1/3 volume of polymer without DNA primer to the PCR sample (i.e. if the initial PCR sample is 300 µL then add 100 µL of 1 wt% polymer) and vortex for 10–20 seconds.
   I. Prior to adding the polymer, save 5–10 µL for quality control (**Raw PCR**).
2. **Native precipitation:** add in 1 volume MeOH to sample and vortex precipitate 10–20 seconds.
3. Incubate for at least 1 minute on ice, then centrifuge at 350–2,000 g (centrifuge at < 4°C) for 5 minutes.
4. Decant or gently pipette the supernatant out.
   I. Save supernatant 1 (**SN1**) for quality control.
5. Resuspend the sample in H_2O to sample volume from step 1 and vortex for 5–10 minutes. Make sure the pellet is perfectly dispersed.

    I. Save 10 µL for quality control as PCR input control for PAGE analysis (**Clean PCR**).
6. **Denaturing step:** add 0.22 volumes of basic denaturing buffer (BDB; 0.2 M NaOH, 2 mM EDTA; final concentration of 44 mM NaOH) to the sample.
    I. For shorter length strands (< 500 nt), vortex 10–20 seconds then incubate for 1 minute.
    II. For mid-length strands (500–1,000 nt), vortex 10–20 seconds then incubate for 5 minutes.
    III. For longer length strands (1,000+ nt), vortex 10–20 seconds then incubate for 10 minutes.
7. **Denaturing precipitation:** add 1 volume of MeOH, vortex 10–20 seconds then incubate for 1 minute on ice.
8. Centrifuge sample at 350–2,000 g (centrifuge at < 4°C) for 5 minutes then transfer supernatant to a fresh centrifuge tube (this supernatant contains strand 1).
    I. Centrifuge supernatant again at 20,000 g (centrifuge at < 4°C) for 5–10 minutes to remove any remaining polymer.
    II. Save 10 µL of supernatant for quality control (**SN2**).
    III. Transfer supernatant carefully to new centrifuge tube and proceed with Part III: Isopropanol precipitation.
9. Add 1 volume of washing solution (1 part 1X TRIS-EDTA, 30 mM NaCl and 1 part MeOH).
10. Vortex for 5–10 seconds then centrifuge at 350–2,000 g (centrifuge at < 4°C) for 5 minutes.
11. Decant or gently pipette the supernatant out.
12. Resuspend the sample in H_2O to sample volume from step 1 and vortex for 5–10 minutes. Make sure the pellet is perfectly dispersed.
13. Proceed with sample to Part II: Strand 2 recovery (UDG/DMEDA strand cleavage).

Part II: Strand 2 recovery (UDG/DMEDA strand cleavage)

1. Prepare the following uracil DNA glycosylase (UDG) reaction to create abasic sites from deoxyuridine bases:
    I. 1 volume of sample (continuation from Part I: Strand 1 recovery step 13)
    II. 10X UDG buffer (final concentration should be 1X)
    III. UDG (0.5 µL per 200 µL sample)
    IV. H_2O as needed to adjust final volume
2. Incubate at 37°C for 15 minutes.

3. Add 10X DMEDA (1 M, adjusted to pH 8–9 with acetic acid). Final concentration should be 1X, adjust with H_2O as needed.
4. Incubate at 37°C for 15 minutes to cleave abasic site.
5. Add 0.04 volumes of 1 M NaCl to the sample to aid in polymer precipitation following DMEDA treatment and vortex 10–20 seconds.
6. Add 1 volume of MeOH to the sample, vortex for 10–20 seconds, and incubate for 1 minute on ice to precipitate.
7. Centrifuge sample at 20,000 g (centrifuge at < 4°C) for 5–10 minutes to remove remaining polymer.
8. Transfer supernatant to fresh centrifuge tube and proceed with Part III: Isopropanol precipitation.
    I. Save 10 µL of supernatant for quality control (**SN3**).

Part III: Isopropanol precipitation

1. Set up the following isopropanol precipitation for both strand 1 and strand 2:
    I. Add 0.1 volumes 3 M sodium acetate (NaOAc).
    II. Add 0.8 volumes isopropanol (iPrOH).
    III. **Optional:** add glycogen as a carrier molecule to improve visibility of precipitated ssDNA (5 µL glycogen per 1 mL of sample).
2. **Optional:** Incubate at -20°C for 1–2 hours.
3. Centrifuge sample at 20,000g (centrifuge at <4°C) for 5–45 minutes and carefully decant supernatant.
    I. We tested centrifugation for 5 minutes with an 89 and 1,000 nt ssDNA and recovered similar yields to 45 minutes of centrifugation.
4. Wash with 70–75% EtOH.
5. Centrifuge for 30 seconds with benchtop microcentrifuge **or** 5 minutes at 20,000 g (centrifuge at <4°C) and decant supernatant.
    I. We tested centrifugation for 30 seconds using a benchtop microcentrifuge with an 89 and 1,000 nt ssDNA and recovered similar yields to 5 minutes of centrifugation with a conventional cooled centrifuge.
6. **Optional:** repeat wash step.
7. Air dry pellet at room temperature or 37°C.
8. Resuspend in desired volume of 1X TE or any other preferred buffer.
9. Nanodrop sample.

Protocol S4: DNA origami folding

Materials

- Tris-EDTA buffer (50 mM Tris, 10 mM EDTA, pH 8.0)
- 60 mM $MgCl_2$
- 1 M $MgCl_2$
- MeRPy-generated ssDNA scaffold (ss-3,315 at 50 nM)
- MeRPy-generated ssDNA scaffold (ss-7,308 at 100 nM)
- 30 nm barrel staple strands and miniscaffold stocks (500 nM per staple strand)
- 20 nm rectangle staple pool core staples (641 nM per staple strand)
- 20 nm rectangle staple pool helper staples (6.25 µM per staple strand)
- 20 nm rectangle staple pool handle staples (8.33 µM per staple strand)
- H_2O

30 nm barrel origami reaction setup, folding, and analysis

1. Mix the following components together:

**30 nm barrel DNA origami:**	**Volume (µL):**
50 mM Tris, 10 mM EDTA, pH 8.0	3.4
60 mM MgCl2	4
ss-3,315 (50 nM)	5.5
Staple + miniscaf stock (500 nM per staple strand)	8
Water	19.1
**Total volume**	**40**

2. Fold the reaction mixture for the 30 nm barrel using the following annealing protocol:
    I. Components were mixed together and annealed over the course of 20 hours (80°C (10 minutes), 55°C → 45°C (18 hours, 1 hour 48 minutes / °C), 45°C → 25°C (1 hour), 4°C (hold)).
3. Samples were analyzed via agarose gel electrophoresis (0.5 x TBE; 11 mM $MgCl_2$; pre-stained with EtBr; resolved at 60 V for 3 hours).

4. Upon confirmation of successful folding, the samples were analyzed by negative staining transmission electron microscopy.

20 nm rectangle origami reaction setup, folding, and analysis

1. Mix the following components together:

**20 nm rectangle DNA origami:**	**Volume (µL):**
50 mM Tris, 10 mM EDTA, pH 8.0	5
1 M MgCl2	0.5
ss-7,308 (100 nM)	5
Core staples	7.8
Helper staples	0.96
Handle staples	3.002
Water	27.738
**Total volume**	**50**

2. Fold the reaction mixture for the 20 nm rectangle using the following annealing protocol:
   I. Components were mixed together and annealed over the course of 5 hours (70°C → 20°C (5 hours, 6 minutes / °C), 4°C (hold)).
3. Samples were analyzed via agarose gel electrophoresis (0.5 x TBE; 11 mM $MgCl_2$; pre-stained with EtBr; resolved at 60 V for 3 hours).
4. Upon confirmation of successful folding, the samples were analyzed by negative staining transmission electron microscopy.

Supplemental Figures

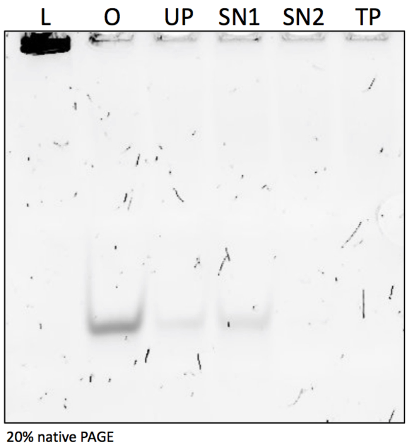

Supplementary Figure S1: Native polyacrylamide gel electrophoresis of tagged primer and supernatants. (L) 1 kb DNA ladder, (O) acrydite tagged primer oligo, (UP) unpurified polymer tagged primer, (SN1) supernatant 1 after first native precipitation clean up, (SN2) supernatant 2 after second native precipitation clean up, (TP) purified polymer tagged primer. Capture yield was quantified by the amount of primer that was not incorporated into the polymer and thus migrated into the native PAGE.

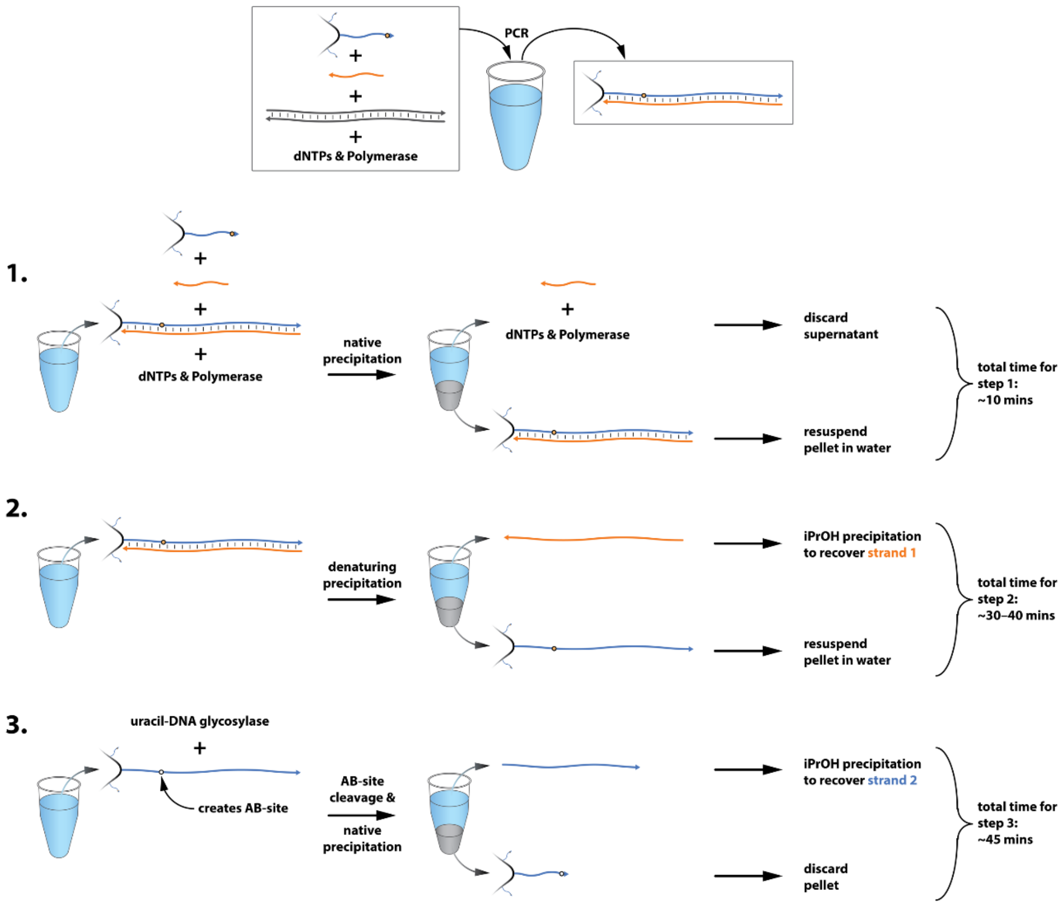

Supplementary Figure S2: General procedure for the recovery of ssDNA. A standard PCR reaction generates the tagged amplicon. (1.) Addition of linear polymer without DNA handles, water and methanol precipitates the tagged amplicon, followed by discarding the supernatant including reverse primer, dNTPs, and polymerase. (2.) Addition of NaOH and water denatures the amplicon and allows the recovery of strand 1 after the addition of methanol. (3.) Incubation of the tagged strand 2 with UDG, subsequent cleavage with DMEDA and precipitation with methanol allows the recovery of strand 2.

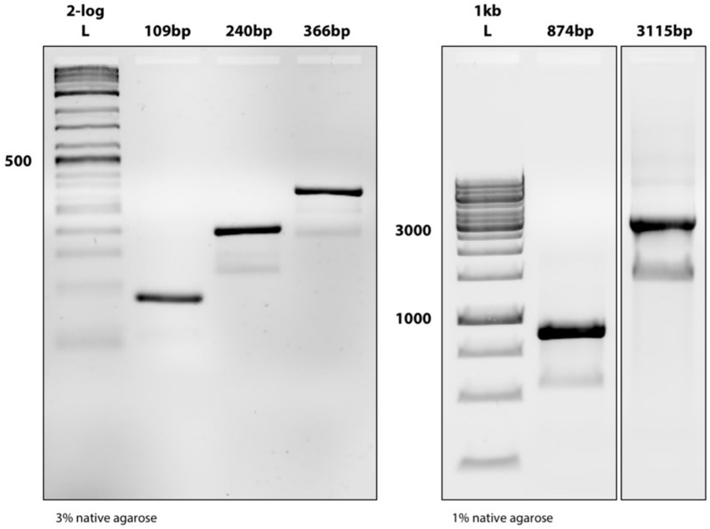

Supplementary Figure S3: Native agarose gel electrophoresis for standard PCR amplicons (dsDNA) on templates used for MeRPy-PCR ssDNA production (shown in Figure 1, see also Supplementary Yield Data).

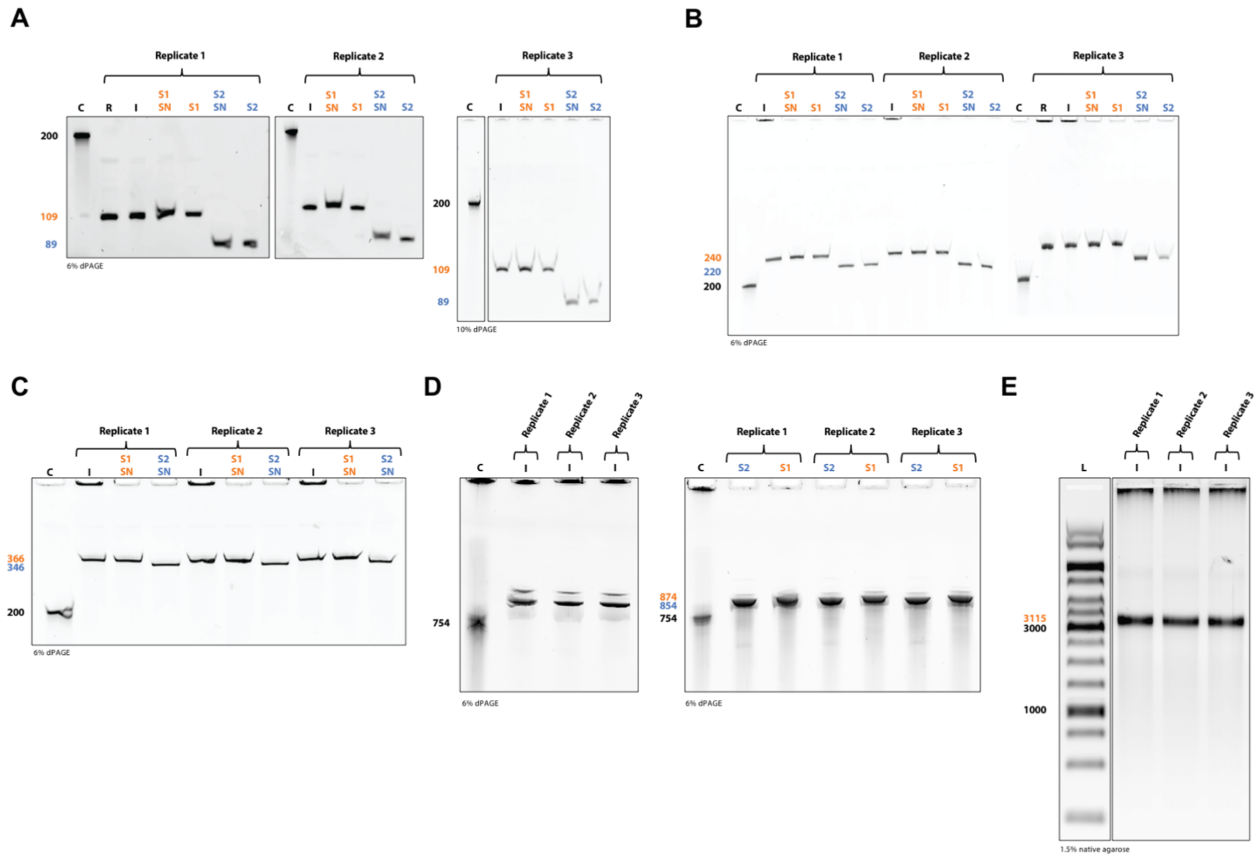

Supplementary Figure S4: ssDNA recovery with 109 – 3,115 bp templates. (**A**) Low range ssDNA recovery with 109 bp template. (c) 200 nt control oligo from IDT, (R) raw MeRPy-PCR reaction, (I) MeRPy-PCR reaction after first native precipitation, (S1 SN) supernatant after denaturing precipitation of strand 1, (S1) strand 1 after iPrOH precipitation, (S2 SN) supernatant after UDG/DMEDA cleavage of strand 2, (S2) strand 2 after iPrOH precipitation. (**B**) Low range ssDNA recovery with 240 bp template. (c) 200 nt control oligo from IDT, (R) raw MeRPy-PCR reaction, (I) MeRPy-PCR reaction after first native precipitation, (S1 SN) supernatant after denaturing precipitation of strand 1, (S1) strand 1 after iPrOH precipitation, (S2 SN) supernatant after UDG/DMEDA cleavage of strand 2, (S2) strand 2 after iPrOH precipitation. (**C**) Low range ssDNA recovery with 366 bp template. (c) 200 nt control oligo from IDT, (I) MeRPy-PCR reaction after first native precipitation, (S1 SN) supernatant after denaturing precipitation of strand 1, (S1) strand 1 after iPrOH precipitation, (S2 SN) supernatant after UDG/DMEDA cleavage of strand 2, (S2) strand 2 after iPrOH precipitation. (**D**) Mid-range ssDNA recovery with 874 bp template. (C) 754 nt control oligo from IDT, (I) MeRPy-PCR reaction after first native precipitation, (S1) strand 1 after iPrOH precipitation, (S2) strand 2 after iPrOH precipitation. (**E**) High range ssDNA recovery with 3,115 bp template. (L) 1 kb ladder, (I) MeRPy-PCR reaction after first native precipitation.

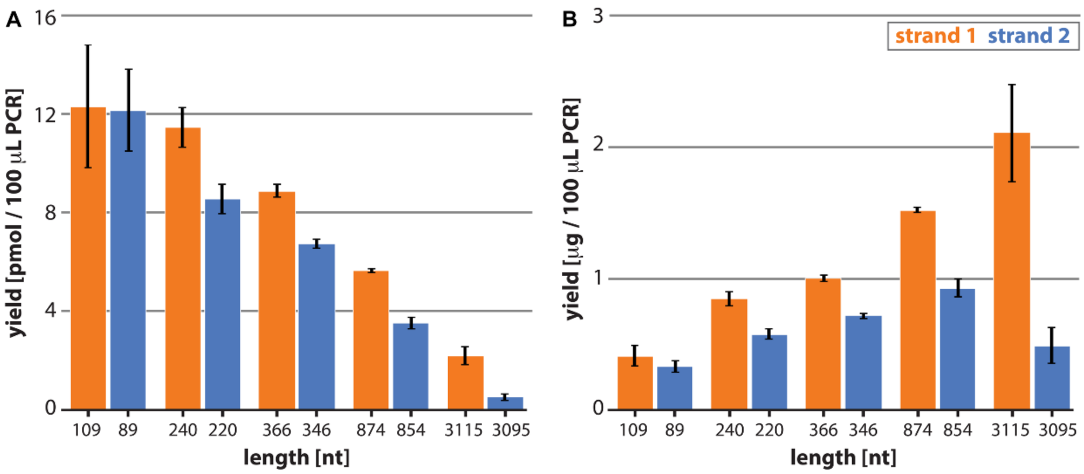

Supplementary Figure S5: Absolute recovery yield for strand 1 and 2 of varying lengths. (**A**) Bar graphs denoting the recovery yield in pmol / 100 µL MeRPy-PCR. (**B**) Bar graphs denoting the recovery yield in µg / 100 µL MeRPy-PCR. Strand recovery yield was determined by nanodrop after the iPrOH precipitation. Data is shown as mean +/- STD (N=3).

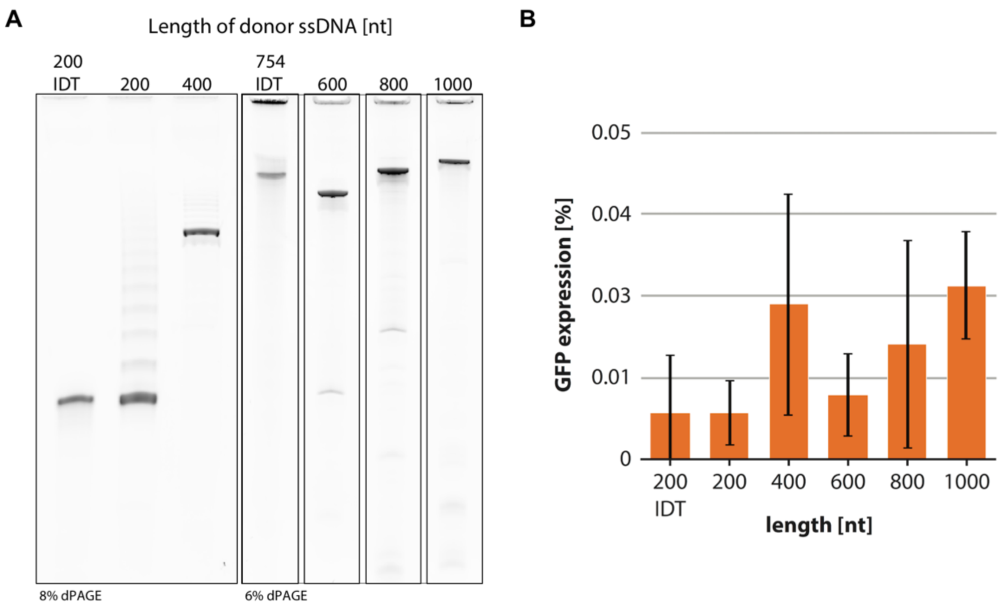

Supplementary Figure S6: (**A**) Denaturing polyacrylamide gel electrophoresis of ssDNA donor oligos (made with MeRPy-PCR) used in CRISPR/Cas9 mediated HDR in human cells. Control lanes on both gels are indicated by 200 and 754 nt ssDNA from IDT. (**B**) Control experiments for CRISPR/Cas9 mediated HDR in human cells. Bar graphs showing GFP expression for cell populations transfected with ssDNA oligo donor alone. Data is shown as mean +/- STD (N=3).

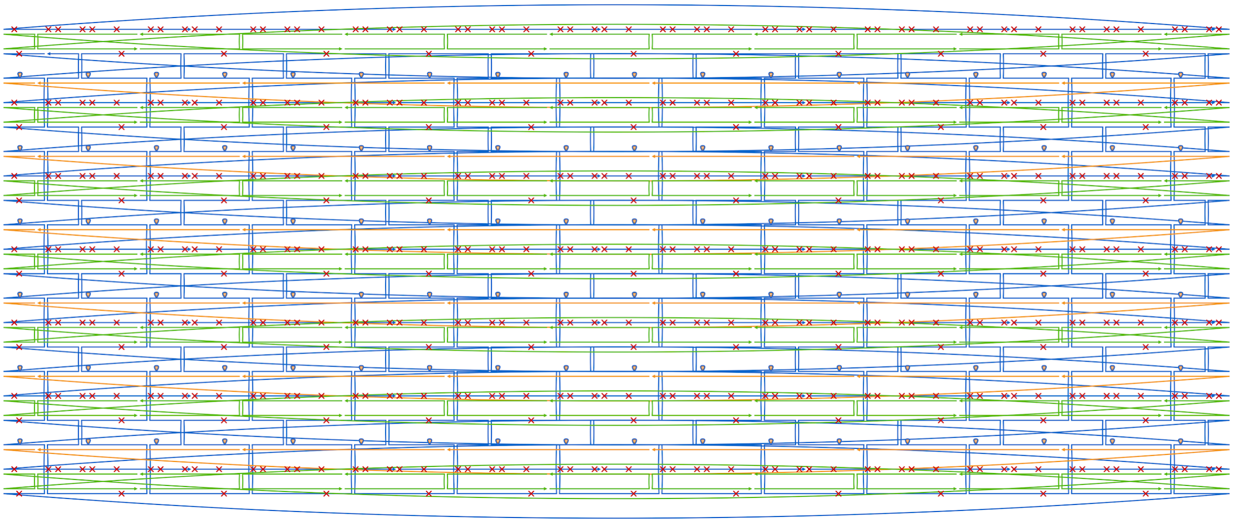

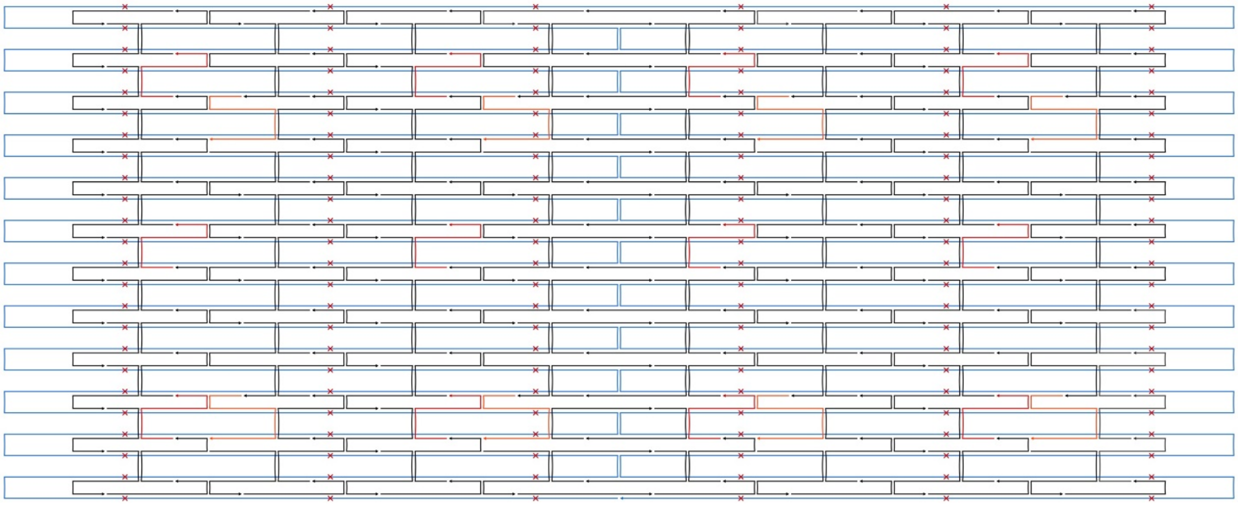

Supplementary Figure S7: DNA origami strand diagram (generated with caDNAno (2)) of 30 nm barrel (top) (3) and the 20 nm rectangle (bottom).

Supplemental Tables

Sample:	Final concentration (μM):	Capture yield (%)
TP - polymer tagged primer used in Figure 1 for all templates	8.823675359	88.23675359

Supplementary Table S1: Densitometry analysis of polymer tagged primer capture yield.

Sample:	Nucleic Acid	Unit	A260 (Abs)	Concentration (µM)	Capture yield (%)
Polymer tagged primer for 200mer	43.2	ng/µl	1.308	5.850804486	58.50804486
Polymer tagged primer for 400mer	20.2	ng/µl	0.613	3.647723784	36.47723784
Polymer tagged primer for 600mer	28.4	ng/µl	0.86	4.61563465	46.1563465
Polymer tagged primer for 800mer	33.7	ng/µl	1.021	4.979093716	49.79093716
Polymer tagged primer for 1,000mer	23.4	ng/µl	0.708	3.45729356	34.5729356
DNA-Origami (30 nm barrel) scaffold polymer tagged primer	29.5	ng/µl	0.89	4.16	83.2
DNA-Origami (20 nm rectangle) scaffold polymer tagged primer	19.2	ng/µl	0.583	2.71	27.1
FISH probes polymer tagged primer	53.2	ng/µl	1.61	8.23	82.3

Supplementary Table S2: Capture yield for MeRPy-primers used in Figure 2. Capture yield was determined by nanodrop analysis (preceded blanking with linear polymer of same wt% without DNA handles).

Sample ID	Nucleic Acid	Unit	Strand length (nt)	MW (g/mol)	Yield (pmol/100 µL PCR)	Yield (µg/100 µL PCR)
200mer for HDR	85.2	ng/µl	200	61530	34.5	2.122785
400mer for HDR	71	ng/µl	370	113830.5	15.575	1.772910038
600mer for HDR	92.8	ng/µl	615	189204.75	14.712	2.783580282
800mer for HDR	137.7	ng/µl	805	247658.25	16.782	4.156200752
1,000mer for HDR	110.5	ng/µl	975	299958.75	13.425	4.026946219
ssDNA scaffold for 30 nm barrel	306.1	ng/µl	3315	1019859.75	1.35	1.376810663
ssDNA scaffold for 20 nm rectangle	222.8	ng/µl	7308	2248306.2	1	2.23
FISH library	281.6	ng/µl	~130	39994.5	70.40966	2.815999147

Supplementary Table S3: Nanodrop yield results of ssDNA donor oligos used in CRISPR/Cas9 mediated HDR, ssDNA scaffold used in DNA origami folding of the 30 nm barrel structure and 20 nm rectangle, and ssDNA probes used in FISH imaging.

Supplemental Notes

Supplementary Note S1: Recovery yield of strand 1 and 2

Data shown in Figure 1 is calculated based on the triplicate MeRPy-PCR results documented in the Supplementary Yield Data excel file. The nanodrop results (calculated into pmol) from the recovered strands 1 and 2 after iPrOH precipitation were compared to the densitometry analysis of the MeRPy-PCR reaction after the first native precipitation. Amplicons generated with MeRPy-PCR are tagged and unable to migrate into the gel. In order to obtain MeRPy-PCR yields, the

samples had to be denatured and separated on denaturing polyacrylamide gel electrophoresis, allowing strand 1 to migrate into the gel. The 3,115 bp MeRPy-PCR amplicon was denatured with formamide and subsequently eluted on a native agarose gel.

Supplementary Note S2: Effect of NaOH denaturation on the recovery yield of strand 2

We have noted that extended exposures of the linear polyacrylamide to the denaturing buffer (NaOH) can cause irreversible polymer damage. Incubation times beyond 10 minutes leads to complete destruction of the polymer and its inability to precipitate from solution with MeOH. We also observed that longer incubations with denaturing buffer lead to decreased strand 2 recovery, likely caused by the additional damage to the polymer. We hypothesize that this is the reason we see diminishing strand 2 recovery with increasing strand length; longer strands require longer denaturing incubations and therefore result in lower recovery yields of strand 2. This hypothesis is in accordance with our results in Figure 1 where we see consistent strand 2 yields for the low-length strands, following the same denaturation protocol. However, we see a decrease in yield for our mid-length strand and an even further decrease in yield for the high-length strand.

Supplementary Note S3: Fluorescence-activated cell sorting (FACS) scatter plots

Live cell population was gated using SSC and FSC to separate debris and singlets. GFP+ gates were set using a transfected control cell population that did not receive the HDR donor and controls were performed with ssDNA oligo donor transfection alone.

WT control

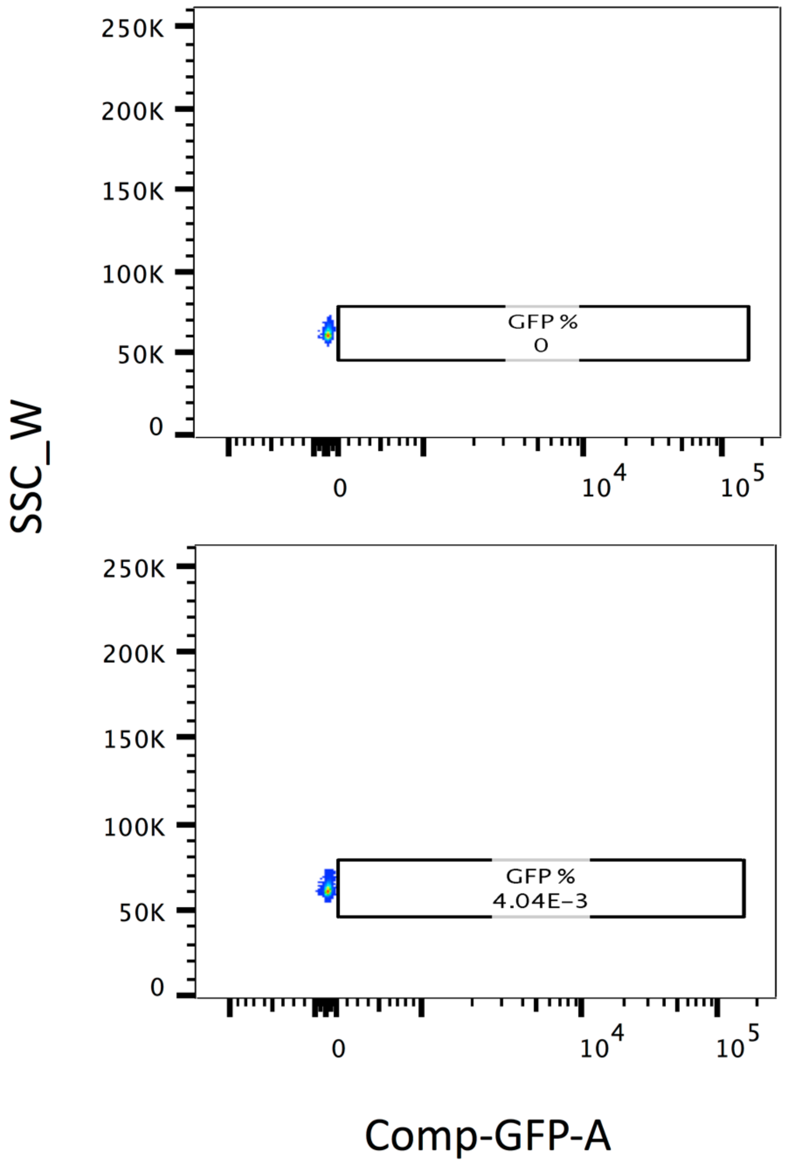

250K
200K
150K
100K
50K
0
GFP %
0
0
10^4
10^5
SSC_W
250K
200K
150K
100K
50K
0
GFP %
4.04E-3
0
10^4
10^5
Comp-GFP-A

IDT 200mer

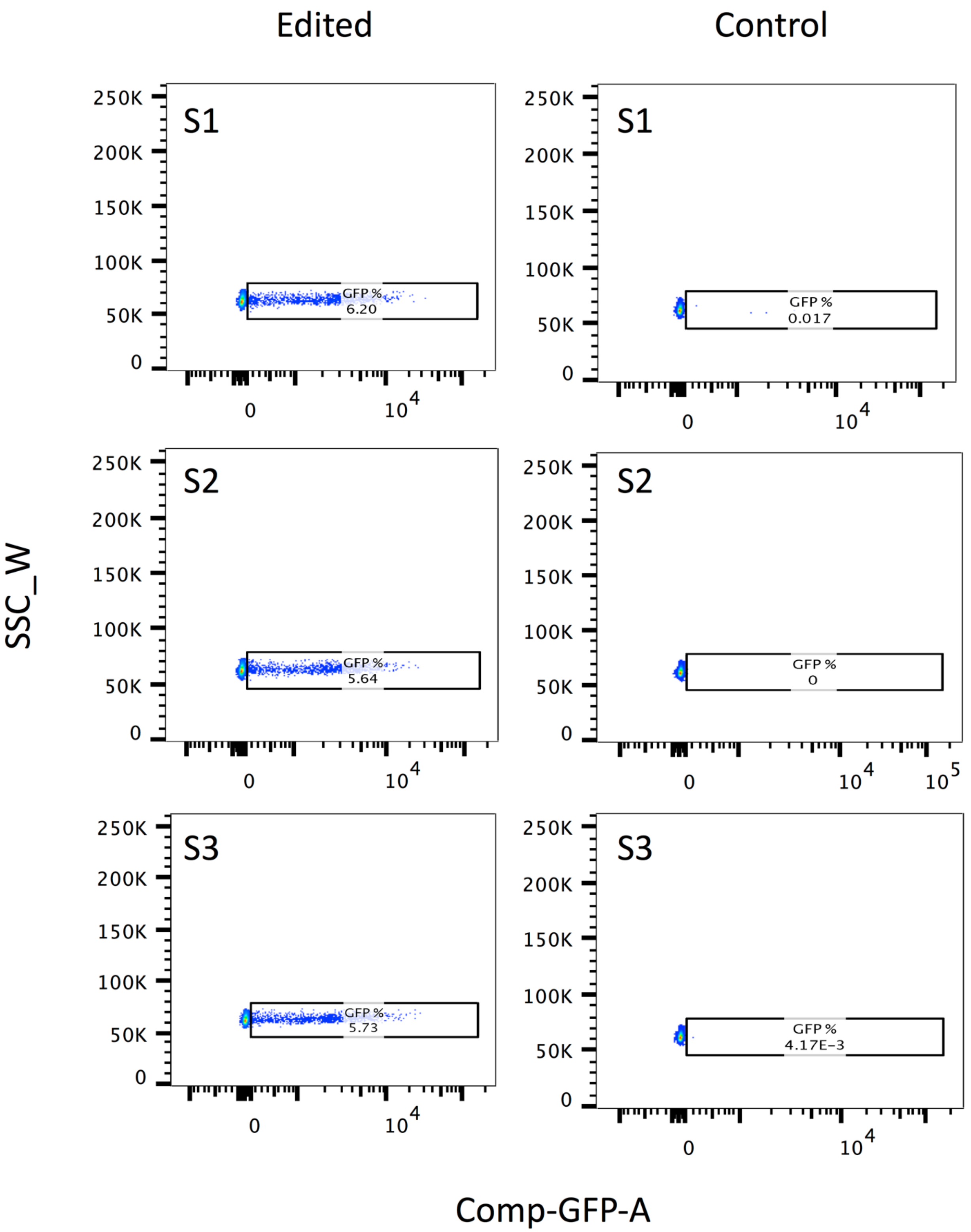

Edited
Control
S1
GFP %
6.20
S1
GFP %
0.017
S2
GFP %
5.64
S2
GFP %
0
S3
GFP %
5.73
S3
GFP %
4.17E-3
250K
200K
150K
100K
50K
0
10^4
10^5
SSC_W
Comp-GFP-A

200mer

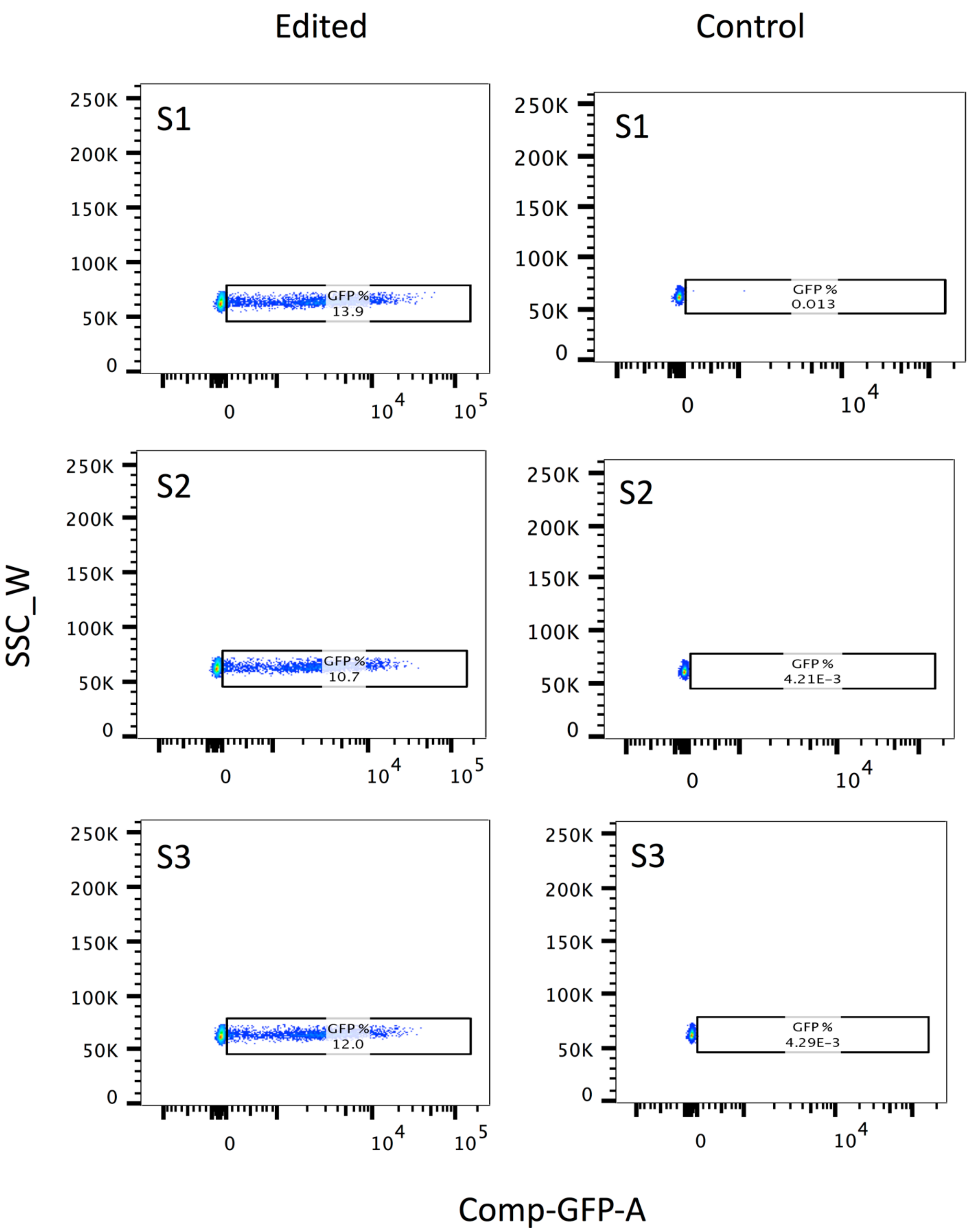
Edited
Control
S1
S2
S3
SSC_W
Comp-GFP-A
GFP %
13.9
10.7
12.0
0.013
4.21E-3
4.29E-3
250K
200K
150K
100K
50K
0
0
10^4
10^5

400mer

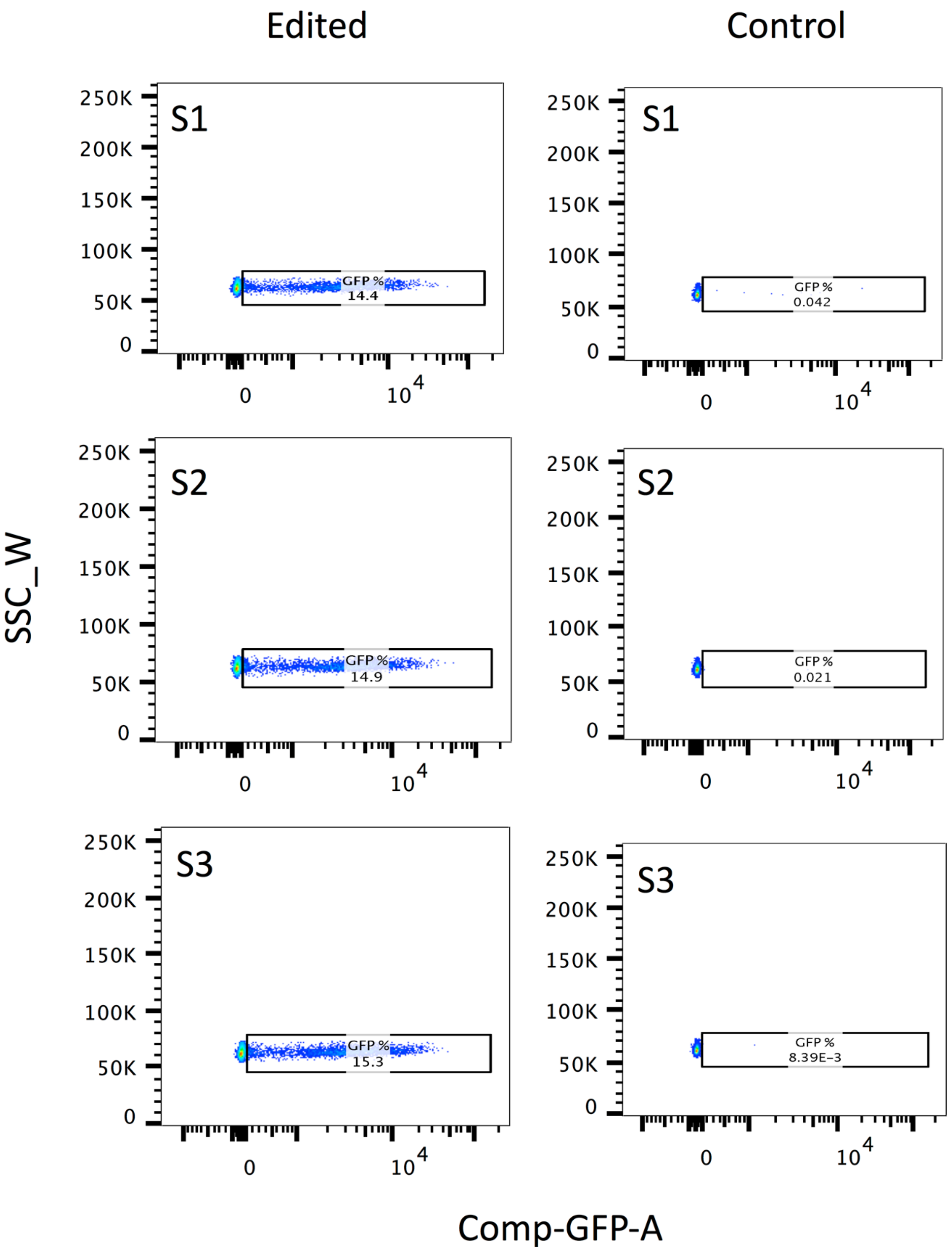
Edited
Control
S1
S2
S3
GFP %
14.4
14.9
15.3
0.042
0.021
8.39E-3
250K
200K
150K
100K
50K
0
10^4
SSC_W
Comp-GFP-A

600mer

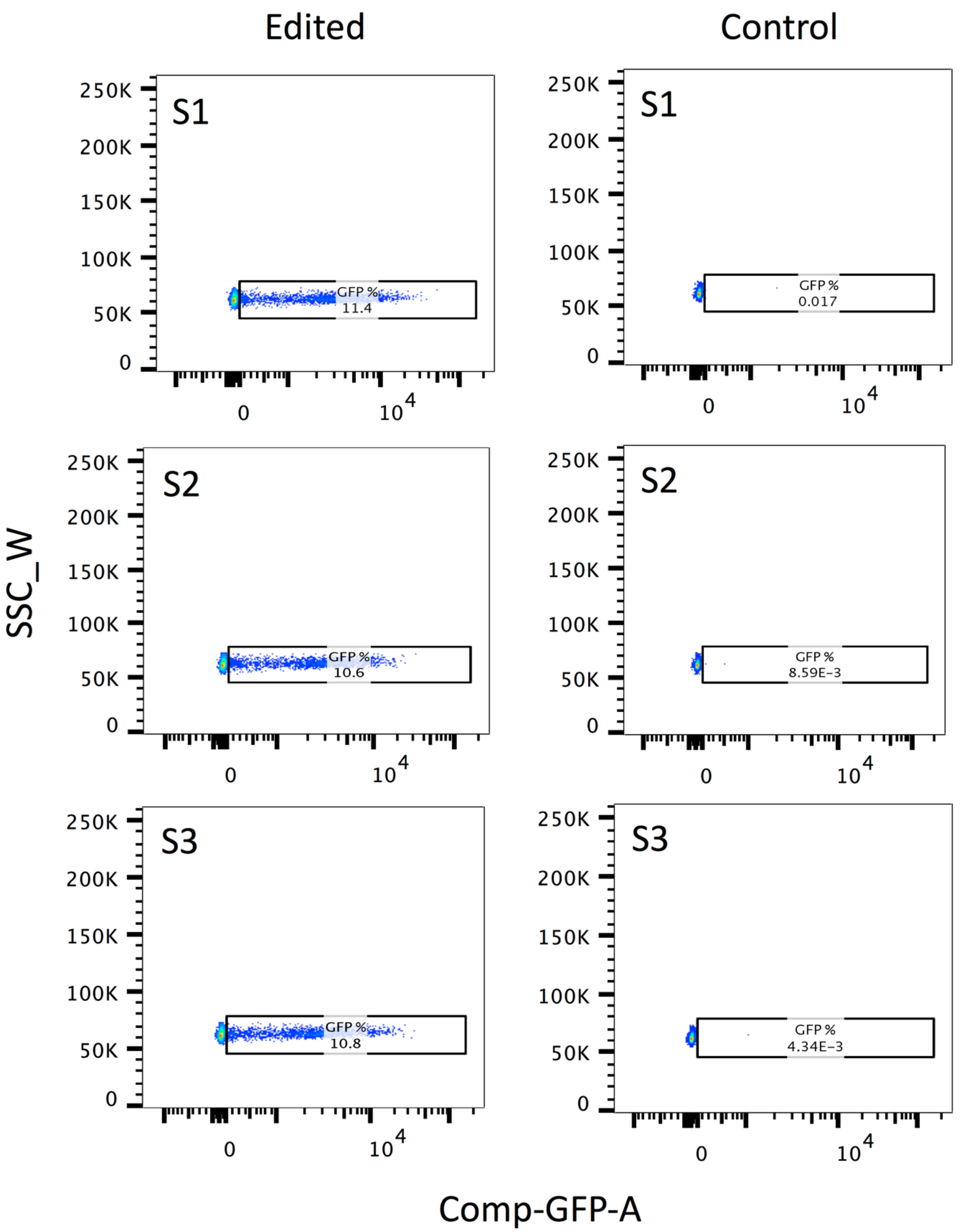

Edited
Control
S1
GFP %
11.4
GFP %
0.017
S2
GFP %
10.6
GFP %
8.59E-3
S3
GFP %
10.8
GFP %
4.34E-3
250K
200K
150K
100K
50K
0
SSC_W
0
10^4
Comp-GFP-A

800mer

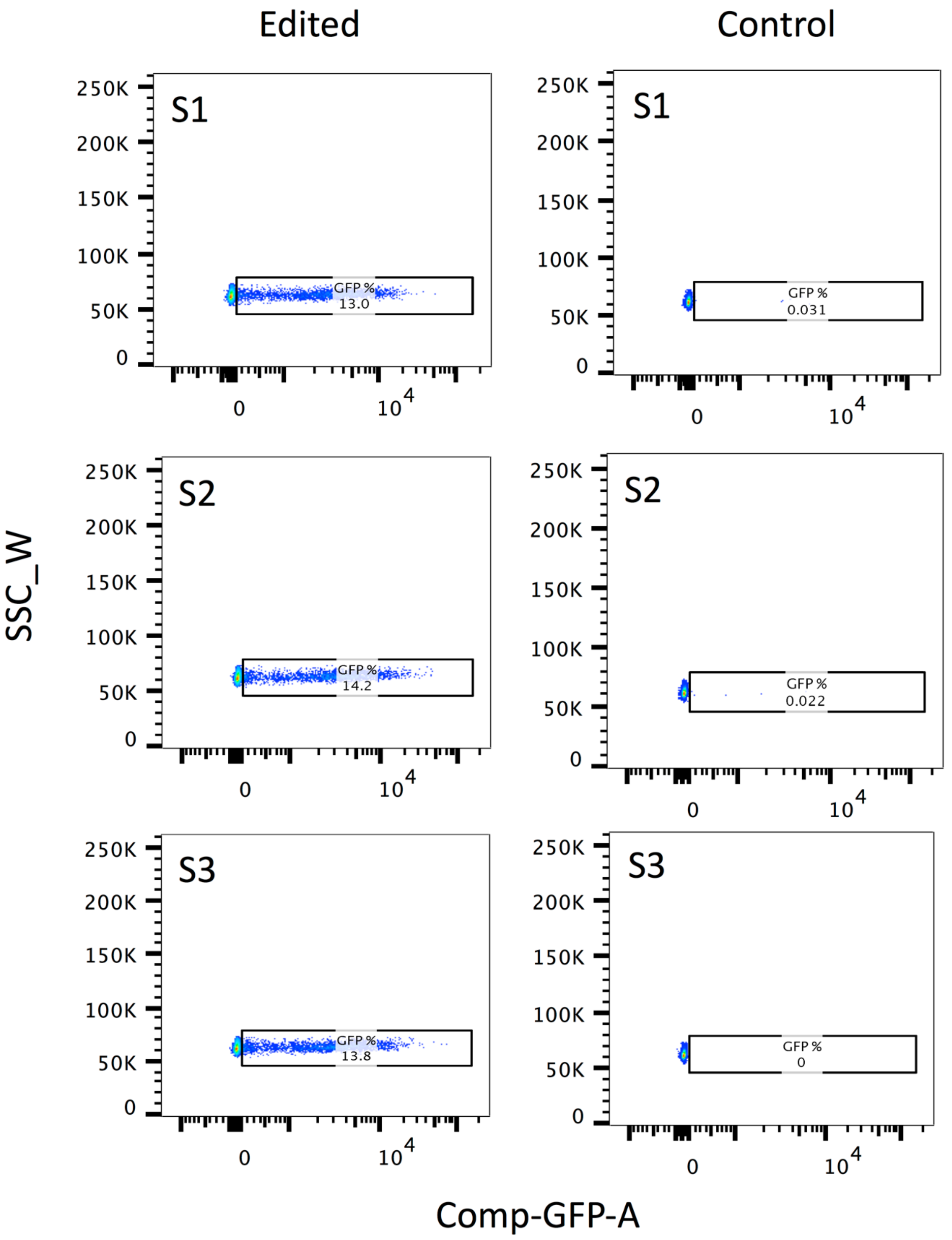

Edited
Control
S1
S2
S3
GFP %
13.0
14.2
13.8
0.031
0.022
0
250K
200K
150K
100K
50K
0
10^4
SSC_W
Comp-GFP-A

1000mer

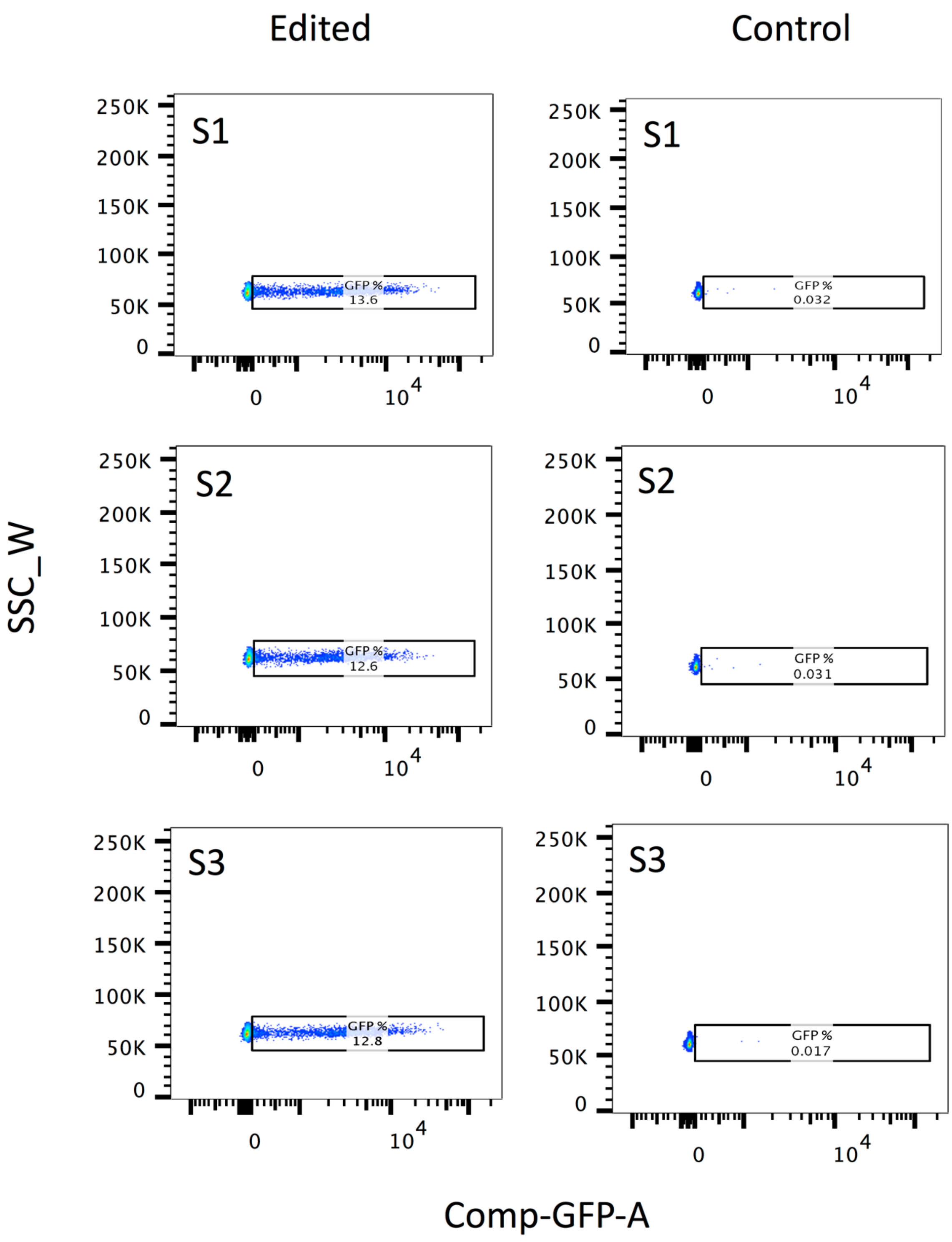
Edited
Control
S1
S2
S3
GFP %
13.6
12.6
12.8
0.032
0.031
0.017
250K
200K
150K
100K
50K
0
10^4
SSC_W
Comp-GFP-A

References

1. Krieg,E. and Shih,W.M. (2018) Selective Nascent Polymer Catch-and-Release Enables Scalable Isolation of Multi-Kilobase Single-Stranded DNA. *Angewandte Chemie International Edition*, **57**, 714–718.

2. Douglas,S.M., Marblestone,A.H., Teerapittayanon,S., Vazquez,A., Church,G.M. and Shih,W.M. (2009) Rapid prototyping of 3D DNA-origami shapes with caDNAno. *Nucleic acids research*.

3. Ponnuswamy,N., Bastings,M.M., Nathwani,B., Ryu,J.H., Chou,L.Y., Vinther,M., Li,W.A., Anastassacos,F.M., Mooney,D.J. and Shih,W.M. (2017) Oligolysine-based coating protects DNA nanostructures from low-salt denaturation and nuclease degradation. *Nature communications*, **8**, 15654.
